# Two Novel Intronic Mutations in the *CSF1R* Gene in Two Families With CSF1R-Microglial Encephalopathy

**DOI:** 10.3389/fcell.2022.902067

**Published:** 2022-05-24

**Authors:** Jiwei Jiang, Wenyi Li, Xiaohong Wang, Zhongli Du, Jinlong Chen, Yaou Liu, Wei Li, Zhonghua Lu, Yanli Wang, Jun Xu

**Affiliations:** ^1^ Department of Neurology, Beijing Tiantan Hospital, Capital Medical University, Beijing, China; ^2^ China National Clinical Research Center for Neurological Diseases, Beijing, China; ^3^ Institute of Translational Medicine, Medical College, Yangzhou University, Yangzhou, China; ^4^ Jiangsu Key Laboratory of Experimental and Translational Non-coding RNA Research, Yangzhou University, Yangzhou, China; ^5^ National Center for Clinical Laboratories, Institute of Geriatric Medicine, Beijing Hospital/National Center of Gerontology, Chinese Academy of Medical Sciences, Beijing, China; ^6^ Division of Neurology, Department of Geriatrics, National Clinical Key Specialty, Guangzhou First People’s Hospital, School of Medicine, South China University of Technology, Guangzhou, China; ^7^ Department of Radiology, Beijing Tiantan Hospital, Capital Medical University, Beijing, China; ^8^ Guangdong Provincial Key Laboratory of Brain Connectome and Behavior, The Brain Cognition and Brain Disease Institute, Shenzhen Institutes of Advanced Technology, Chinese Academy of Sciences, Shenzhen, China

**Keywords:** CSF1R (colony stimulating factor 1 receptor), microglia, dementia, gene, mutation, magnetic resonance imaging

## Abstract

**Objective:** To describe two novel heterozygous splicing variants of the *CSF1R* gene responsible for CSF1R-microglial encephalopathy in two unrelated Han Chinese families and further explore the relationship between the pathological and neuroimaging findings in this disease.

**Methods:** The demographic data, detailed medical history, and clinical manifestations of two unrelated Han families with CSF1R-microglial encephalopathy were recorded. Some family members also underwent detailed neuropsychological evaluation, neuroimaging, and genetic testing. The probands underwent whole-exome sequencing (WES) or next-generation sequencing (NGS) to confirm the diagnosis. The findings were substantiated using Sanger sequencing, segregation analysis, and phenotypic reevaluation.

**Results:** Both families presented with a dominant hereditary pattern. Five of 27 individuals (four generations) from the first family, including the proband and his sister, father, uncle, and grandmother, presented with cognitive impairments clinically during their respective lifetimes. Brain magnetic resonance imaging (MRI) depicted symmetric, confluent, and diffuse deep white matter changes, atrophy of the frontoparietal lobes, and thinning of the corpus callosum. The proband’s brother remained asymptomatic; brain MRI revealed minimal white matter changes, but pseudo-continuous arterial spin labeling (pCASL) demonstrated a marked reduction in the cerebral blood flow (CBF) in the bilateral deep white matter and corpus callosum. Seven family members underwent WES, which identified a novel splice-site heterozygous mutation (c.2319+1C>A) in intron 20 of the *CSF1R* gene in four members. The proband from the second family presented with significant cognitive impairment and indifference; brain MRI depicted symmetric diffuse deep white matter changes and thinning of the corpus callosum. The proband’s mother reported herself to be asymptomatic, while neuropsychological evaluation suggested mild cognitive impairment, and brain MRI demonstrated abnormal signals in the bilateral deep white matter and corpus callosum. NGS of 55 genes related to hereditary leukodystrophy was performed for three members, which confirmed a novel splice-site heterozygous mutation (c.1858+5G>A) in intron 13 of the *CSF1R* gene in two members.

**Conclusions:** Our study identified two novel splicing mutation sites in the *CSF1R* gene within two independent Chinese families with CSF1R-microglial encephalopathy, broadening the genetic spectrum of CSF1R-microglial encephalopathy and emphasizing the value of pCASL for early detection of this disease.

## Introduction

In 1936, van Bogaert and Nyssen described a Belgian family who presented with adult-onset orthochromatic leukodystrophy and dementia, whose histopathological characteristics included diffuse myelin and axonal loss, accompanied by pigmented macrophages and termed the condition pigmentary orthochromatic leukodystrophy (POLD) (Van and Nyssen, 1936). In 1984, Axelsson et al. found 17 members from a Swedish family, who presented with various combinations of psychiatric, neurological, and somatic symptoms, with pathological features similar to those of POLD, since they were characterized by the loss of axons and myelin sheaths, and accumulation of astrogliosis, pigmented macrophages, and axonal spheroids in the cerebral white matter, which lent the disease its name of hereditary diffuse leukoencephalopathy with spheroids (HDLS) (Axelsson et al., 1984). Over the past 20 years, several unrelated families presenting with inherited adult-onset leukodystrophy and pathological neuroaxonal spheroids and pigmented glia have been reported (Köhler et al., 2018). These conditions have been known by a series of names ranging from the initial terms POLD and HDLS to the more recent term adult-onset leukoencephalopathy with axonal spheroids and pigmented glia (ALSP) (Konno et al., 2018a; Guo and Ikegawa, 2021).

A mutation in the colony-stimulating factor-1 receptor (*CSF1R*) gene has been recently identified in some families with pathological diagnoses of the above-mentioned conditions (Konno et al., 2018a; Lynch et al., 2019), which provides genetic evidence that these conditions actually belong to a vast phenotypic spectrum of a single disease entity. The *CSF1R* gene encodes the CSF1R protein (a transmembrane tyrosine kinase receptor), which is mainly expressed in the microglia in the brain and mediates the proliferation, differentiation, and survival of the microglia (Marzan et al., 2021). *CSF1R* mutation can result in amino acid changes in the tyrosine kinase domain (TKD) and microglial dysfunction, culminating in rapidly progressive dementia (Tsai et al., 2021). Moreover, recent studies have demonstrated that *CSF1R* mutation can affect brain structure (cerebral white matter change) and cause various brain dysfunctions, including early-onset cognitive impairment, behavioral changes, parkinsonism, seizures, and depression (Konno et al., 2018b; Tian et al., 2019). Thus, we suggest the term CSF1R-microglial encephalopathy, which encompasses genetic, pathological, and clinical characteristics, to describe and unify the diverse phenotypic spectrum of *CSF1R* mutation.

Since the discovery of *CSF1R* mutations in families with CSF1R-microglial encephalopathy, a total of 115 mutation sites have been identified worldwide (Wang et al., 2021; Wu et al., 2022). The majority of these mutations were missense and were found in Japanese, European, and American populations (Konno et al., 2017). Presently, neither the functional consequences of all known *CSF1R* mutations nor the full genetic spectrum of mutations causing CSF1R-microglial encephalopathy have been fully elucidated. The significant variability in the initial clinical manifestations can easily result in underdiagnosis or misdiagnosis as Alzheimer’s disease (AD), multiple sclerosis, frontotemporal dementia, and other leukoencephalopathies. Herein, we reported two novel splicing mutation sites (c.2654+1G>A and c.1858+5G>A) in the intron of the *CSF1R* in two unrelated Han Chinese families and analyzed the relationship between the pathological and neuroimaging characteristics, with the aim of expanding the genetic and radiological spectrum of CSF1R-microglial encephalopathy.

## Materials and Methods

### Ethics

The study was approved by the Institutional Review Board of Beijing Tiantan Hospital of Capital Medical University (KY-2021-028-01). All individuals involved in this study provided written informed consent for genetic analysis before enrollment.

### Participants and Data Collection

Information on sex, age, age of onset (if applicable), and clinical manifestations were collected from all members of both unrelated families. Three individuals from the first family and two individuals from the second family underwent MRI using 3.0-T devices with the following sequences: T1-weighted imaging (T1WI), T2-weighted imaging (T2WI), fluid-attenuated inversion recovery (FLAIR), diffusion**-**weighted imaging (DWI), T1-contrast enhancement, angiography, and pseudo-continuous arterial spin labeling (pCASL).

### Genetic Analysis

Whole-exome sequencing (WES) and a hereditary leukodystrophy-targeted next-generation sequencing (NGS) panel (55 genes including *ABCD1*, *ARSA*, *CSF1R*, *EIF2B2*, *GJC2*, *PEX1*, *POLR3A*, *POLR3B*, *PSAP*, *TREX1*, *EIF2B1*, *EIF2B3*, *EIF2B4*, *EIF2B5*, *PLP1*, *GFAP*, *ASPA*, *MLC1*, *HEPACAM*, *YP27A1*, *GBE1*, *RNASEH2A*, *RNASEH2B*, *RNASEH2C*, *SAMHD1*, *ADAR*, *LMNB1*, *SLC17A5*, *FUCA1*, *FAM126A*, *GALC*, *L2HGDH*, *DARS2*, *EARS2*, *SUMF1*, *GJA1*, *RNASET2*, *HSD17B4*, *ACOX1*, *SCP2*, *ALDH3A2*, *SOX10*, *MFSD8*, *HSPD1*, *MPV17*, *PEX10*, *PEX13*, *PEX5*, *PEX6*, *POLR1C*, *SDHA*, *SDHB*, *TMEM187*, *NOTCH3,* and *HTRA1*) were conducted to discover the causative variants in the patients from the first and second families, respectively. Briefly, genomic DNA was isolated from peripheral leukocytes using the DNA Isolation Kit (Blood DNA Kit V2, CWE 9600, CoWin Biosciences, China). Subsequently, the qualified DNA samples were fragmented into 200–300 base pairs (bp). DNA libraries were prepared using the KAPA Library Preparation Kit KR0453 (Kapa Biosystems, United States*) following the manufacturer’s instructions. The libraries were estimated with the Qubit dsDNA HS Assay kit (Invitrogen, Q32851) and finally sequenced on the Illumina NovaSeq 6000 platform (Illumina, Inc., United States) using paired-end 200-bp reads. The sequencing data were filtered and aligned with the human reference genome (GRCh37/hg19) using the BWA Aligner (http://bio-bwa.sourceforge.net/). The quality scores were estimated. Consensus calling for single-nucleotide polymorphisms (SNPs) and insertions and deletions (indels) was performed using the Genome Analysis Toolkit. Each of the called variants was annotated using ANNOVAR (annovar.openbioinformatics.org/en/latest/) using several public databases [such as 1000 Genomes Project, Exome Aggregation Consortium (ExAC), gnomAD, ESP6500, CCDS, RefSeq, and Ensembl]. The annotation content was composed of the variant position, variant type, allele frequency, and conservation prediction, which can help locate mutations corresponding to diseases.

The variants identified in our patients were first filtered against the 1000 Genomes Project database for a minor allele frequency (MAF) ≥ 1%, and ExAC hom AC ≥ 3. The variants obtained *via* this analysis were further filtered according to the co-segregation, genetic model, and MAF <1% against three databases (1000 Genomes Project, EAS, ExAC, and gnomAD). The SNPs and indels occurring in the exons and canonical splice sites were analyzed using multiple computational software programs to predict the effects of these variants. The Professional Version of the Human Gene Mutation Database (HGMD) was searched to determine if the variants were reported previously. Finally, the selected variants were classified according to the American College of Medical Genetics and Genomics (ACMG) and Association for Molecular Pathology (AMP) guidelines (Tavtigian et al., 2018). Sanger sequencing was conducted to validate the familial segregation of the variants.

## Results

### Clinical Characteristics of the First Family

**FIGURE 1:**
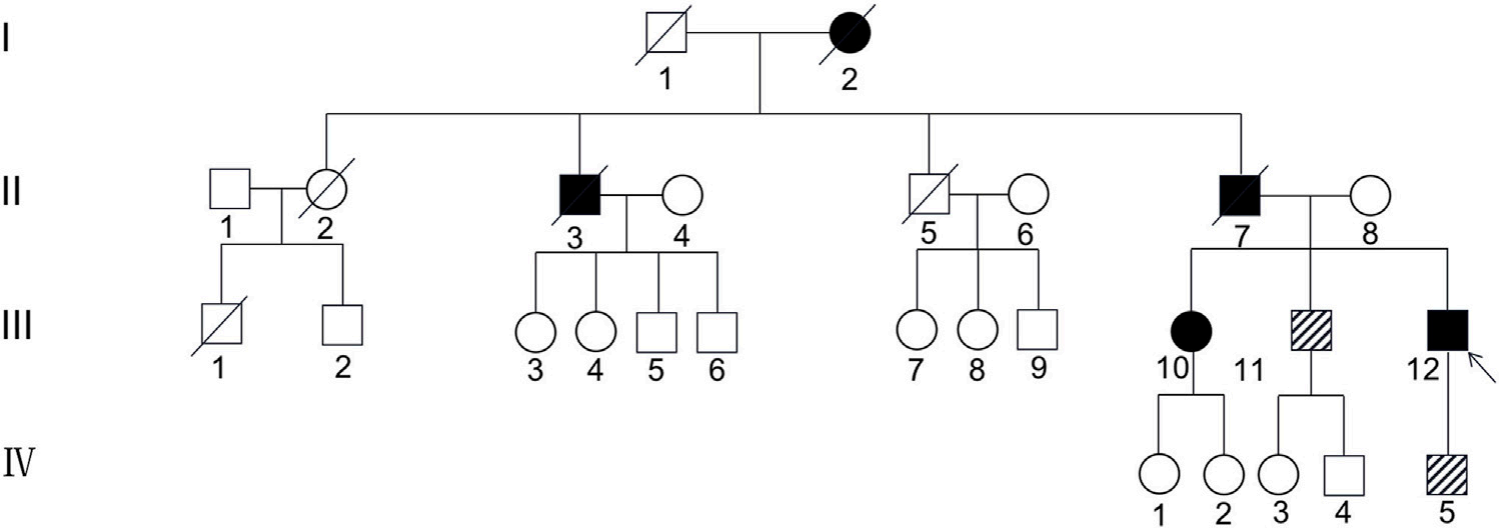
Pedigree of the first Han Chinese family with CSF1R-microglial encephalopathy. The Roman numeral to the left of the pedigree denotes the generation. Women are represented by circles, and men by squares. The arrow indicates the proband (patient III-12). Black symbols represent affected patients, while white symbols represent unaffected individuals or at-risk individuals of unknown phenotype. Oblique symbols represent that the patients with *CSF1R* mutation were asymptomatic.

The first family is a nonconsanguineous family of Han Chinese origin that spanned four generations and consisted of 27 members. The family pedigree is depicted in Figure 1. Five of the 27 individuals, including the proband, his sister, father, uncle, and grandmother, presented with similar cognitive impairments clinically. However, it was not possible to conduct genetic testing for the father, uncle, and grandmother since they had died before the study was conducted. *CSF1R* mutation was detected in four of the seven members who underwent genetic testing, including the proband, his sister, brother, and son. The brother (45 years) and son (17 years) of the proband had the same *CSF1R* mutation but remained asymptomatic.

#### Proband Ⅲ-12 of the First Family

The proband was a 43-year-old man with a high school education. He first experienced dizziness and constant numbness in the distal portion of the bilateral upper limbs at the age of 39 years. He developed cognitive and psychiatric impairment 5 months after onset, characterized by executive dysfunction, attention deficit, and indifference. The above-mentioned clinical symptoms gradually worsened, and he developed speech problems, including slow speech and anomia and weakness in the bilateral lower limbs by the age of 41 years. The cognitive decline and indifference were exacerbated at 42 years of age, and the slow and slurred speech and speaking problems also progressed. Furthermore, he developed gait disturbance and rigidity and required a wheelchair for mobility. He was admitted to Beijing Tiantan Hospital in November 2020. He denied having a history of medication or substance abuse. There was no medical history of hypertension, diabetes mellitus, coronary heart disease, and traumatic brain injury. Neurological examination demonstrated mild dysarthria and non-fluent aphasia; impaired cognition with respect to orientation, memory, calculation, comprehension, and executive ability; and left facial nerve and hypoglossal nerve palsy. The muscle strength of all four extremities was grade 4 with slightly increased muscle tone. A postural tremor was absent. Hyperreflexia was observed in the knees and ankles bilaterally, with a bilateral positive Babinski sign. Both upper limbs exhibited superficial hypoesthesia. He also walked with a slow and shuffling gait. Poor bilateral finger–nose and heel–knee coordination was also observed.

**FIGURE 2:**
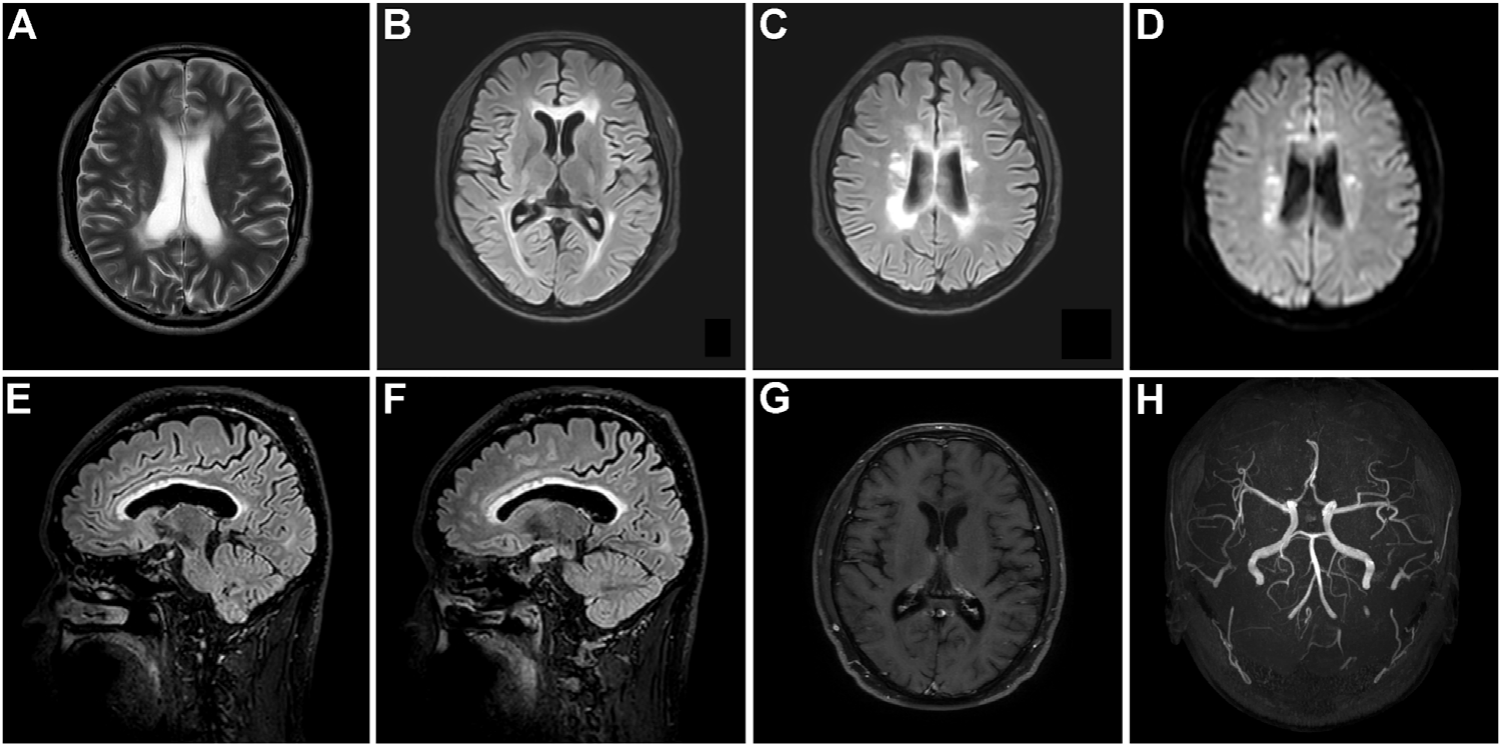
Brain MRI of the proband with CSF1R-microglial encephalopathy in the first family. Bilateral confluent and diffuse deep white matter hyperintensities involving periventricular areas, corona radiate and the corpus callosum, and moderate diffuse atrophy of the bilateral frontoparietal lobes can be seen on brain axial T2-weighted imaging **(A)**, fluid-attenuated inversion recovery (FLAIR) **(B,C)** and diffusion-weighted imaging **(D)**. Sagittal brain FLAIR shows abnormal hyperintensities and thinning of the corpus callosum, and extensive cortical atrophy **(E,F)**. No obvious abnormalities can be found in the Gadolinium contrast-enhanced MRI **(G)** and cerebral arteries on magnetic resonance angiography **(H)**.

The proband’s Mini-Mental State Examination (MMSE) score was 15/30, with substantial deterioration in orientation, calculation, and executive ability. The Montreal Cognitive Assessment (MoCA) score was 6/30 with obvious deficits in orientation, memory, calculation, comprehension, and executive ability. The results of laboratory tests, including routine blood analyses; liver, renal, and thyroid function; ion concentration; erythrocyte sedimentation rate; homocysteine, vitamin B, and C-reactive protein levels; and immunological biomarkers of rheumatic disease were unremarkable. He also tested negative for anti-aquaporin-4, anti-myelin oligodendrocyte glycoprotein, and autoimmune encephalitis-related antibodies (NMDAR, AMPAR1/2, LGI1, CASPR2, GABAR, and GAD65). Brain MRI revealed relatively symmetric confluent and diffuse white matter hyperintensities that markedly affected the bilateral periventricular areas, corona radiata, and corpus callosum; prominent frontal and parietal lobe atrophy; and thinning of the corpus callosum (Figures 2A–F). Gadolinium-based contrast-enhanced MRI did not demonstrate significant enhancement changes (Figure 2G). Magnetic resonance angiography did not reveal obvious abnormalities in the cerebral arteries (Figure 2H).

**FIGURE 3:**
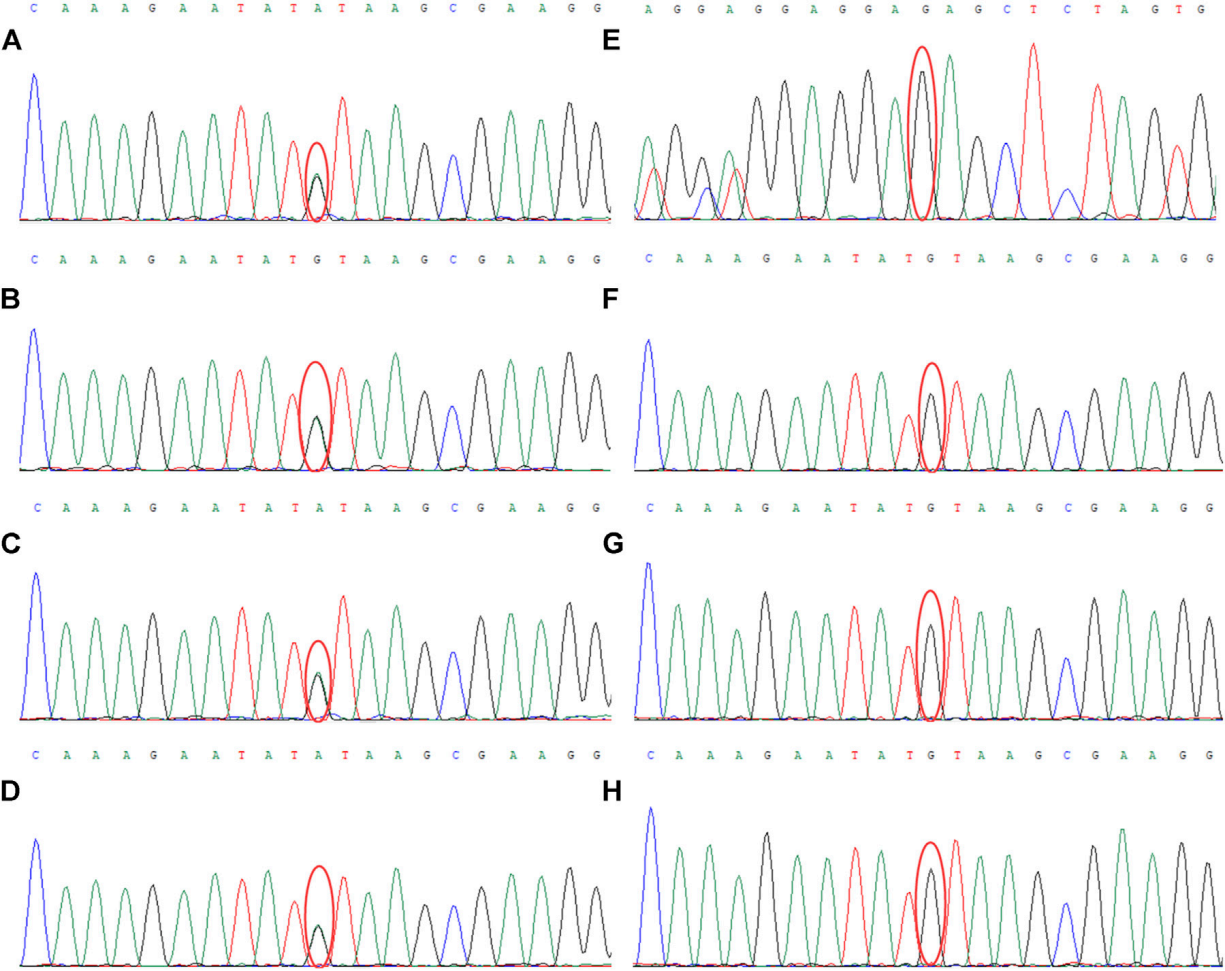
Sanger sequencing chromatogram of *CSF1R* gene mutation analysis of seven family members in the first family. Sequencing chromatograms identified a novel heterozygous mutation in the *CSF1R* gene on chromosome 5q32: one is a splicing mutation (c.2654+1G>A) in intron 20 of *CSF1R* in the proband **(A)**, patient Ⅲ-10 **(B)**, patient Ⅲ-11 **(C)**, and patient Ⅳ-5 **(D)**, and the other is an indel mutation in exon 22 (c.2833_2835delGAG) of *CSF1R* **(E)**. No mutation of the *CSF1R* gene can be found in patient Ⅱ-8 **(F)**, patient Ⅳ-1 **(G)**, and patient Ⅳ-2 **(H)**.

The results of WES demonstrated a novel heterozygous mutation in the *CSF1R* gene located on chromosome 5q32 that consisted of a splicing c.2654+1G>A mutation in intron 20 (Figure 3A) and an indel mutation in exon 22 (c.2833_2835delGAG) (Figure 3E). These data have been deposited in the Sequence Read Archiver repository (https://www.ncbi.nlm.nih.gov/sra/PRJNA780236). These mutation sites were not found in the ExAC, Human Gene Mutation Database, or 1000 Genomes Project. Mutation Taster, PROVEAN, and FATHMM-indel predicted that mutation c.2833_2835delGAG was a polymorphism (probability, 0.999), neutral (score, −2.10 > −2.5), and neutral (score, 0.365), respectively. However, Mutation Taster, FATHMM-XF, and CADD predicted that mutation c.2654+1G>A was deleterious (probability, 0.99), pathogenic (probability, 0.992), and deleterious (score, 33), respectively. dbscSNV classified the splicing c.2654+1G>A mutation as “disease-causing,” with an adaptive boosting score of 0.999 and random forest score of 0.934. Mutation Taster, NetGene2, and Human Splicing Finder predicted that the mutation might alter the WT donor site, with a high probability of interrupting normal splicing. Thus, the splicing c.2654+1G>A mutation was predicted to be possibly pathogenic (PVS1+PM2), while the indel mutation (c.2833_2835delGAG) was of uncertain significance (PM2+PM4), according to the ACMG/AMP guidelines. The c.2654+1G>A mutation was not found in the Genome Aggregation Database, while the c.2833_2835delGAG mutation was present in this database. There are six individuals carrying this indel mutation, from 40 to 80 years old, none of whom have any clinical symptoms. Therefore, we speculated that the splicing mutation (c.2654+1G>A) in intron 20 was more significant than the indel mutation (c.2833_2835delGAG) in this family and disrupted the function of *CSF1R*, resulting in CSF1R-microglial encephalopathy.

This patient was diagnosed with CSF1R-microglial encephalopathy according to the 2018 diagnostic criteria for adult-onset leukoencephalopathy with ASLP due to *CSF1R* mutation after collating the clinical manifestations and neuroimaging and genetic findings (Konno et al., 2018b). Memantine (10 mg/day) was administered to improve cognitive function. However, the patient’s condition continued to deteriorate rapidly. He became bedridden and could not perform purposeful movements at the follow-up examination performed in July 2021. He could not communicate with others and developed urinary and bowel incontinence.

#### Patient Ⅲ-10 of the First Family

**FIGURE 4:**
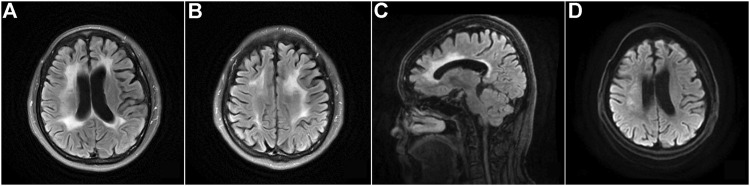
Brain neuroimaging of the proband’s sister (patient Ⅲ-10) with CSF1R-microglial encephalopathy. Bilateral diffuse hyperintense lesions affecting the periventricular white matter, corpus callosum, and deep areas of the frontoparietal lobes on the brain axial FLAIR **(A,B)** and DWI **(D)**. Sagittal brain FLAIR shows abnormal hyperintensities and thinning of the corpus callosum, and extensive cortical atrophy **(C)**.

This patient, the proband’s sister, was a 49-year-old woman with a middle school education. Although the symptoms of cognitive impairment were similar to those of the proband, the other clinical symptoms diverged slightly. She presented with conspicuous memory decline and fatigue at 46 years of age. She was indifferent and developed depression, although her speech was fluent and clear. The above-mentioned symptoms progressed 1 year after onset, in addition to the onset of bradykinesia and rigidity. However, she did not exhibit sensory abnormalities. Neurological examination revealed fluent speech but low volume of voice; Gerstmann syndrome; and impaired cognition with respect to orientation, memory, calculation, comprehension, and executive ability. The cranial nerves were normal. The muscle strength and tendon reflexes of all four extremities appeared normal, with a slight increase in the muscle tone of both upper limbs. Her gait was slow, and she found it a little difficult to turn around. The Babinski sign was negative bilaterally. Her MMSE and MoCA scores were 12 and 6, respectively. The Hamilton Depression Rating Scale score was 23, and the Hamilton Anxiety Rating Scale score was 15, suggesting mild depression and anxiety, respectively. Brain MRI revealed bilateral diffuse hyperintense lesions affecting the deep white matter of the frontoparietal lobes and corpus callosum, as well as **e**xtensive cortical atrophy (Figure 4). WES demonstrated a mutation at the same site in the *CSF1R* gene as that in the proband (Figure 3B). Her condition deteriorated to a prominent decline in cognition, indifference, and rigidity 30 months after onset.

#### Patient Ⅲ-11 of the First Family

**FIGURE 5:**
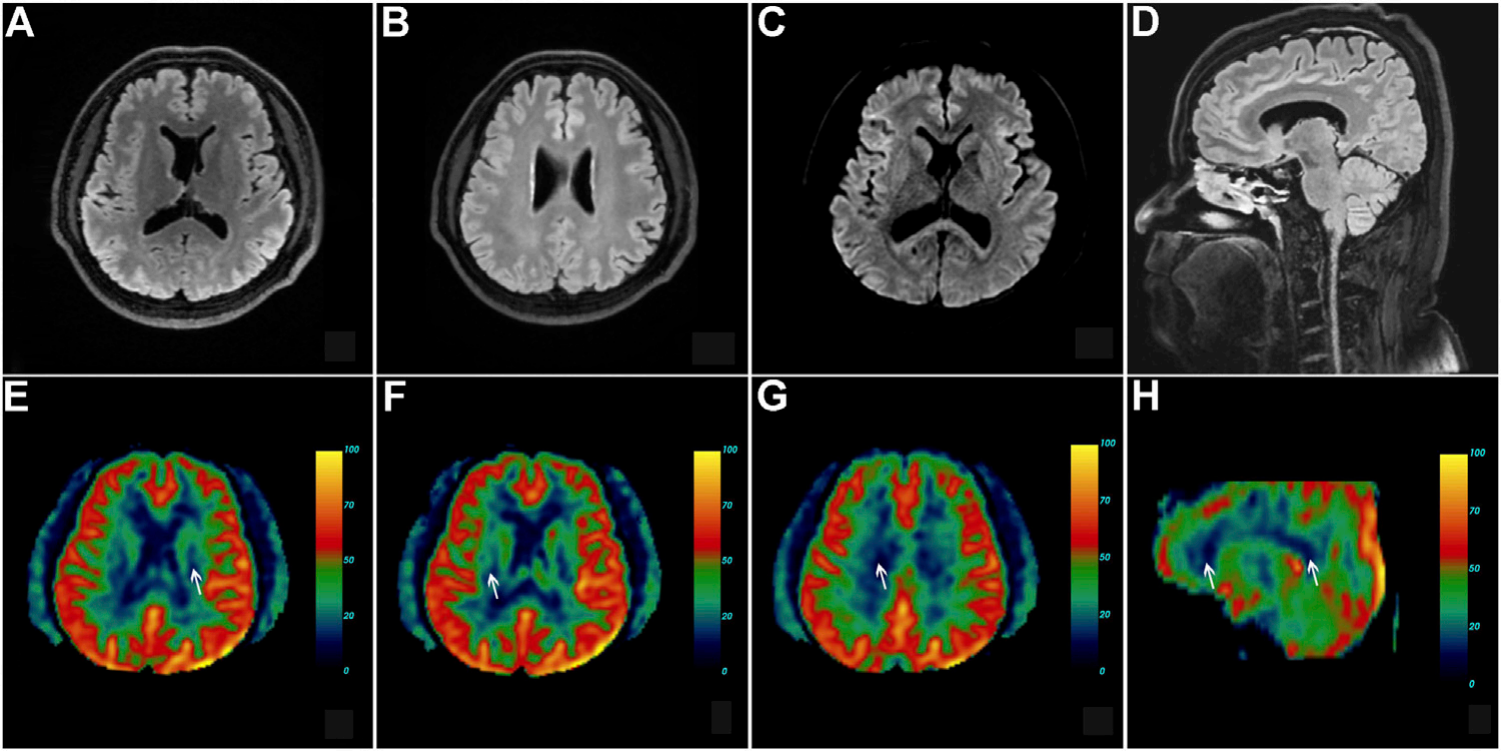
Brain neuroimaging of the proband’s brother (patient III-11) in the first family. Brain FLAIR demonstrates few slight hyperintensities in the periventricular white matter **(A,B)** and a focal high signal in the body of the corpus callosum **(D)**. There is no abnormal signal in the axial diffusion-weighted imaging **(C)**. Brain pseudo-continuous arterial spin labeling revealed a marked reduction of the cerebral blood flow (CBF) in the bilateral deep and subcortical white matter of frontoparietal lobes. The point value of the CBF in the left periventricular area is 15.3 ml/100 g/min **(E)**, the point value of the CBF in the right periventricular area is 18.6 ml/100 g/min **(F)**, the point value of the CBF in the subcortical deep white matter of parietal lobe is 8.6 ml/100 g/min **(G)**, and the value of CBF in the anterior corpus callosum is 16.8 ml/100 g/min **(H)**, while that in the splenium of corpus callosum is 13.5 ml/100 g/min.

This patient, the proband’s brother, was a 45-year-old man with a bachelor’s degree. He did not present with any cognitive, psychiatric, motor, and sensory dysfunction until November 2020. The findings of the neurological examination were unremarkable. Structural brain MRI demonstrated bilateral scattered slightly hyperintense lesions affecting the periventricular white matter and a high-signal intensity dot in the trunk of the corpus callosum (Figures 5A–D). Brain pCASL revealed a conspicuous reduction in CBF in the deep white matter of the frontal and parietal lobes and corpus callosum (Figures 5E–H). WES demonstrated a mutation at the same site in the *CSF1R* gene as that in the proband (Figure 3C). This individual remained asymptomatic at the follow-up examination conducted in September 2021.

### Clinical Characteristics of the Second Family

**FIGURE 6:**
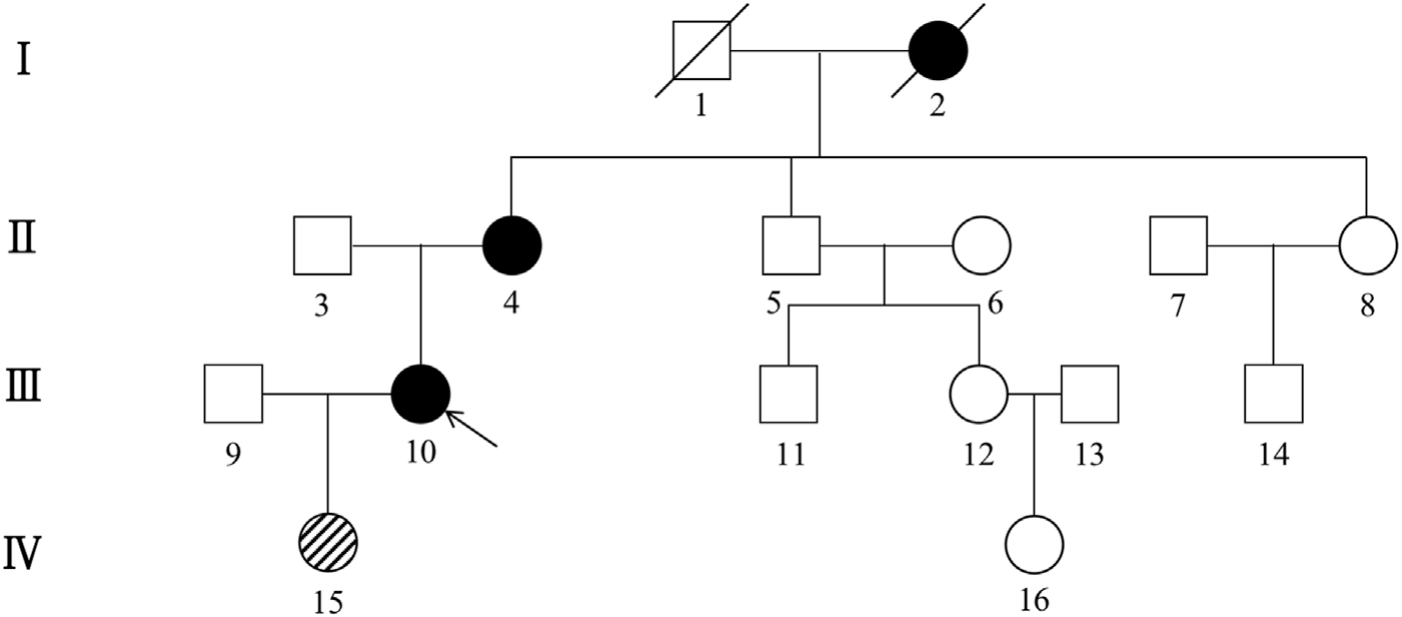
Pedigree of the second Han Chinese family with CSF1R-microglial encephalopathy. The Roman numeral to the left of the pedigree denotes the generation. Women are represented by circles, and men by squares. The arrow indicates the proband (patient III-10). Black symbols represent affected patients, while white symbols represent unaffected individuals or at-risk individuals of unknown phenotype. Oblique symbols represent that the patients with *CSF1R* mutation were asymptomatic.

The second family is a nonconsanguineous family of Han Chinese origin spanning four generations and comprising 16 members. The family pedigree is depicted in Figure 6. Only the proband and her grandmother exhibited obvious cognitive impairment, but genetic testing was impossible since her grandmother was deceased. The daughter (22 years) and the mother (73 years) of the proband had the same *CSF1R* mutation but remained asymptomatic.

#### Proband Ⅲ-10 of the Second Family

The proband was a 46-year-old woman with a middle school education. She first experienced deterioration in memory and location orientation and psychiatric impairment at the age of 44 years. She forgot her way home and presented with indifference and forced laughter. The MMSE and MoCA scores were 26 and 20, respectively, suggesting mild cognitive impairment. One year later, her cognition worsened sharply, involving memory, executive function, orientation, calculation, and visuospatial reactions. She experienced an extreme deterioration in comprehension, indifference, disinhibition, and urinary incontinence. However, her speech was fluent and clear. She did not have a resting tremor or dyskinesia. The neurological examination revealed fluent speech and impaired cognition with respect to orientation, memory, calculation, comprehension, and executive ability. The cranial nerves were normal. The muscle strength, tendon reflexes, and muscle tone of all four extremities appeared normal. Her gait was normal, without tremor, rigidity, bradykinesia, or postural instability. She denied having a history of hypertension, diabetes mellitus, coronary heart disease, traumatic brain injury, and medication or substance abuse. The MMSE and MoCA scores deteriorated to 2 and 1, respectively at her age of 46 years. The results of laboratory tests demonstrated an elevation in the thyroid-stimulating hormone levels at 9.356 μIU/ml (normal range: 0.38–4.34), but the free triiodothyronine and tetraiodothyronine levels were normal.

**FIGURE 7:**
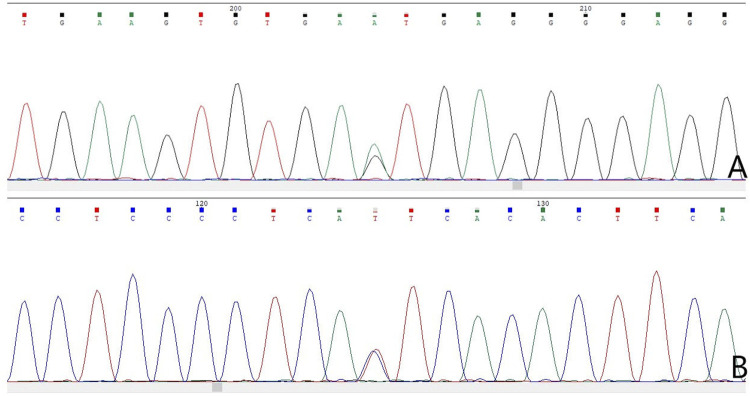
Sanger sequencing chromatogram of *CSF1R* gene mutation analysis of four family members in the second family. Sequencing chromatograms identified a novel heterozygous mutation in the *CSF1R* gene on chromosome 5q32: a splicing mutation (c.1858+5G>A) in intron 13 of *CSF1R* in the proband **(A)** and patient Ⅱ-4 **(B)**.

Brain MRI revealed confluent and diffuse signal abnormalities in the bilateral periventricular areas, corona radiata, and corpus callosum, and prominent thinning of the corpus callosum (Figures 7A–D).

**FIGURE 8:**
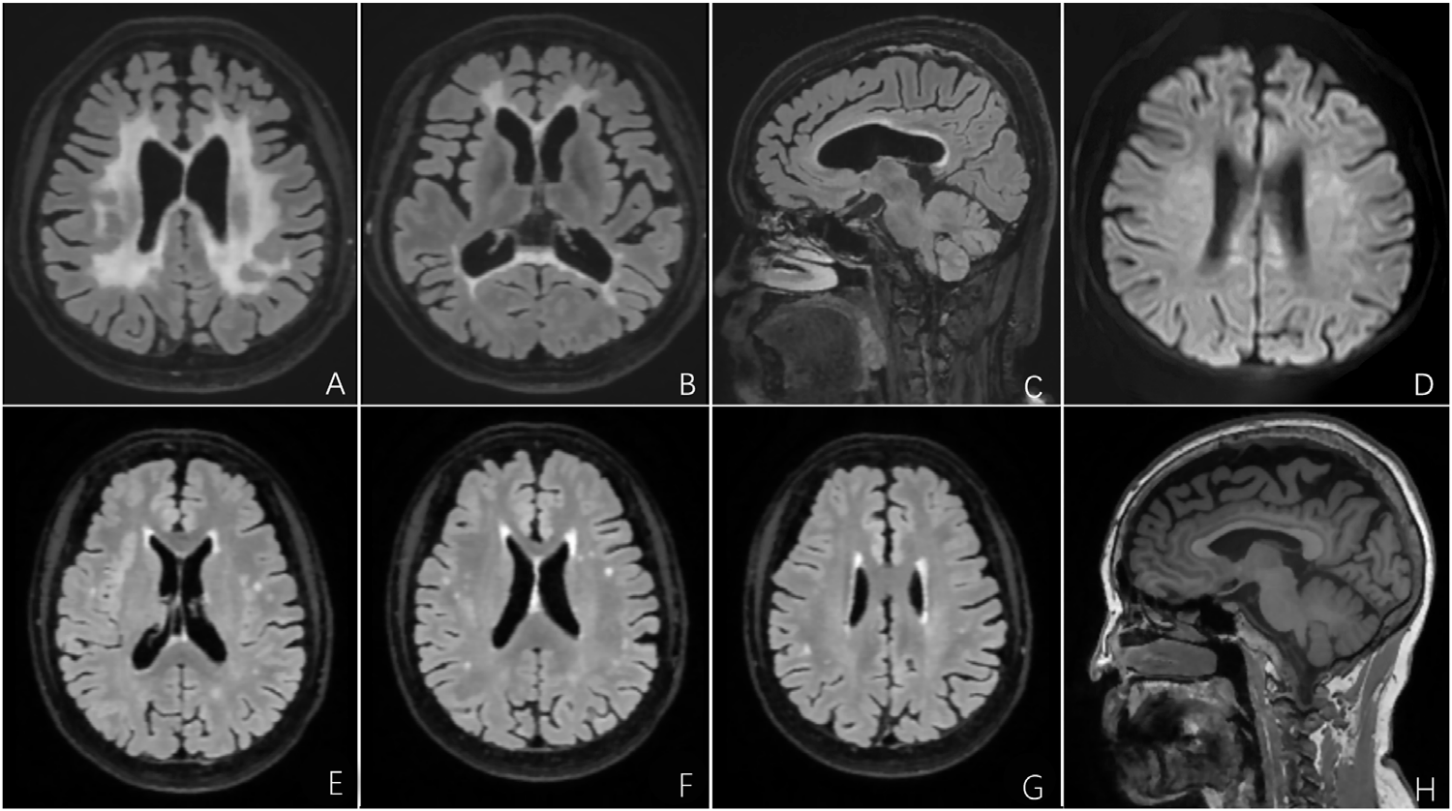
Brain MRI of the proband and her mother with CSF1R-microglial encephalopathy in the second family. Bilateral confluent and diffuse deep white matter hyperintensities involving periventricular areas, corona radiate and the corpus callosum on the FLAIR imaging **(A**,**B)** and DWI **(D)**. Sagittal brain FLAIR shows abnormal hyperintensities and thinning of the corpus callosum, and extensive cortical atrophy **(C)**. Bilateral confluent deep white matter hyperintensities involving periventricular areas, corona radiate and the trunk of corpus callosum on the FLAIR imaging **(E**–**G)**. Sagittal brain T1-weighted imaging shows significant thinning of the corpus callosum, and extensive cortical atrophy **(H)**.

The results of NGS and Sanger sequencing identified a novel heterozygous splicing c.1858+5G>A mutation in intron 13 of the *CSF1R* gene (Figure 8A). The mutation site was not found in the ESP6500, HGMD, dbSNP and 1000 Genomes Project databases. Mutation Taster, FATHMM-XF and CADD predicted this mutation as deleterious (probability, 0.99), pathogenic (probability, 0.993), and deleterious (score, 33), respectively. The programs NetGene2 and Human Splicing Finder predicted that the mutation may alter the WT donor site, most likely disturbing the normal splicing. No other symptoms-related variants have been identified. Thus, the splicing c.1858+5G>A mutation was predicted to be possibly pathogenic (PVS1+PM2) according to the ACMG/AMP guidelines.

#### Patient Ⅱ-4 of the Second Family

This patient, the proband’s mother, was a 73-year-old woman with a junior middle-school education. She did not present with cognitive, psychiatric, and motor dysfunction until January 2022. The neurological examination findings were unremarkable except for impaired memory and calculation ability. However, the MMSE score was 22, and the MoCA score was 19. Moreover, the Hopkins Verbal Learning Test revealed that she could recall 5, 3, and 2 words at the immediate, 5-min, and 20-min time points, respectively, which indicated significant immediate and delayed memory impairment and suggested mild cognitive impairment. Brain MRI demonstrated bilateral scattered hyperintensities affecting the periventricular white matter and trunk of the corpus callosum, significant atrophy of the frontoparietal lobes, and thinning of the corpus callosum (Figures 8E–H). The results of NGS and Sanger sequencing identified a mutation at the same site in the *CSF1R* gene as that in the proband (Figure 7B).

## Discussion

Since the discovery of *CSF1R* mutations in families with CSF1R-microglial encephalopathy, 115 mutation sites have been identified worldwide, including 93 missense mutations, four nonsense mutations, four insertions or deletions, seven frameshift mutations, and 14 splice-site mutations (Tian et al., 2019; Wu et al., 2022). The CSF1R is a protein tyrosine kinase receptor that consists of five immunoglobulin-like domains in the extracellular ligand-binding portion, a single transmembrane domain, a juxtamembrane domain, a kinase insertion domain, and two intracellular TKDs (Tarale and Alam, 2022). Approximately 95% of *CSF1R* gene mutations are located in the TKD of CSF1R encoded by exons 12–21, especially exons 18 and 19 (Leng et al., 2019; Zhuang et al., 2020; Chitu et al., 2021). No disease-associated mutations have been discovered yet in exon 16 (Tian et al., 2019). Of the 14 splicing mutations documented in *CSF1R*, three occurred in intron 12, six in intron 18, and one each in introns 13, 16, 17, 20, 21 (Tian et al., 2019; Wu et al., 2022). All of these variant sites lie within the TKD, affecting the mRNA level and leading to a partial loss of CSF1R protein function (Leng et al., 2019). Our study found two novel splicing mutations of *CSF1R* within two unrelated Han Chinese families: c.2654+1G>A of intron 20 and c.1858+5G>A in intron 13, enlarging the genetic spectrum of CSF1R-microglial encephalopathy. These two novel splicing intronic mutations also lay within the TKD, consistent with previous studies (Leng et al., 2019; Tian et al., 2019). Moreover, multiple software predicted that these two variants might alter the WT donor site, interrupting normal splicing, resulting in a high likelihood of protein inactivation, despite the lack of protein model and functional verification. Functional examination of the identified mutations is being further explored.

The CSF1R is activated upon ligand engagement, which then undergoes receptor dimerization that leads to autophosphorylation of the tyrosine residues (Hume et al., 2020), triggering multiple signal transduction pathways that regulate the survival, proliferation, differentiation, and activation of cells of the macrophage lineage (Muñoz-Garcia et al., 2021). The CSF1R is mainly expressed on the surface of microglia in the central nervous system (CNS) and affects its function in immune and inflammation reactions, synaptic development, and myelin maintenance (Prinz et al., 2019). Diffuse white matter destruction is the histological hallmark of CSF1R-microglial encephalopathy, entailing the loss of axons and myelin, thinning of the corpus callosum, calcification, axonal swelling (“spheroids”), and accumulation of pigmented macrophages (microglia in the CNS), which corresponds to the loss of normal physiologic function of the microglia (Tian et al., 2019). Previous studies have demonstrated quantitative and distributional alterations of the microglia in the brains of patients with HDLS. Tada et al. found that the number of microglia and levels of microglia-associated proteins were significantly decreased in HDLS (Tada et al., 2016), and the impairment in CSF-1 signaling decreased the longevity of the microglia rather than their proliferative ability (Elmore et al., 2014; Tada et al., 2016). Interestingly, the distribution of microglia exhibits stark differences in HDLS: the cells were significantly reduced in number in layers three to four of the frontal cortex and cerebellar molecular layer, whereas patchy microgliosis was evident in the deeper cortex and cerebral white matter (Tada et al., 2016). Studies on adult zebrafish with *CSF1R* mutation also found that *CSF1R* haploinsufficiency led to reduced microglial density and aberrant distribution of microglia: they were partially or completely absent in the normal-appearing gray matter and white matter, while activated microglia were accumulated in the deep brain regions (Oyanagi et al., 2017; Oosterhof et al., 2018). Accordingly, an animal study has demonstrated that the white matter in higher-order brain regions of the CNS (such as the frontal lobe) may be uniquely dependent on microglial CSF-1 signaling (Erblich et al., 2011). In the deeper cortex and cerebral white matter, the activated microglia were accumulated in the relatively preserved areas but not in the affected areas and exhibited abnormal morphologies and spatial restriction due to *CSF1R* mutation (Tada et al., 2016). These pathological findings characterized by the region-specific distribution of the activated microglia were consistent with the structural brain MRI findings of the current and previous studies (Mao et al., 2020; Mickeviciute et al., 2022), i.e., significant abnormal signals in the deep white matter of the frontoparietal lobe and corpus callosum, revealing the potential relationship between the number and functional changes in the microglia induced by *CSF1R* mutation and deep white matter degeneration.

Interestingly, both probands from the two unrelated families exhibited bilateral persistent hyperintensity in the periventricular white matter on DWI. Previous studies also showed that hyperintense signals on DWI sequences could persist for several months to years in patients with *CSF1R* mutation, consistent with our findings (Bender et al., 2014; Onder et al., 2020). The mechanism underlying diffusion restriction in patients with *CSF1R* mutation remains unknown. Restricted diffusion was suggested to be the manifestation of either high-grade myelin edema (cytotoxic edema) accompanied by leukodystrophic loci or spots of demyelination (Martinez-Saez et al., 2012), which is congruent with the pathological features such as microglia activation-mediated myelin damage and axonal cell swelling. Semi-quantitative immunohistochemistry and quantitative transcriptomic profiling in patients with HDLS has revealed that *CSF1R* mutations can result in the loss of microglial homeostatic phenotype (M0) in the white matter (Kempthorne et al., 2020). Inhibition of CSF1R has been shown to lead to a partial loss of CSF1 signaling, and a phenotypic shift in the microglia from anti-inflammatory/homeostatic to pro-inflammatory/activated (Han et al., 2022). It is speculated that the signal intensity may arise from fluid trapped between the degenerating layers of myelin sheaths (Tokumaru et al., 2021). The selective vulnerability of the white matter and persistent diffusion restriction on brain MRI can be explained by the pathological characteristics of *CSF1R* mutation: *CSF1R* mutation leads to microglial loss and abnormal distribution, and activates the microglia into the inflammatory phenotype (M1), and eventually causes demyelination, axonal loss and swelling, which precedes white matter degeneration. This finding is in line with the notion that *CSF1R* mutation-induced leukoencephalopathy is a primary microglial disorder and reemphasizes the feasibility of the nomenclature “CSF1R-microglial encephalopathy.”

Since the discovery of *CSF1R* mutations in 2012, conditions such as HDLS, ASLP, brain malformations, and skeletal dysplasia have been considered as part of the varied phenotypic spectrum of a unified disease (Rademakers et al., 2011). In this study, the proband of the first family presented with cognitive impairment, psychiatric symptoms, pyramidal signs, and parkinsonism. His affected sister (patient Ⅲ-10) and the proband of the second family exhibited cognitive impairment and indifference. All of their manifestations are in accordance with the five core features described in the diagnostic criteria for ASLP induced by *CSF1R* mutation established in 2018 (Konno et al., 2018b). Although patient Ⅱ-4 of the second family did not have any specific complaint or clinical symptoms, the neuropsychological evaluation suggested mild cognitive impairment and significant memory decline. These findings are consistent with previous studies (Jin et al., 2015; Zhuang et al., 2020), which found that cognitive impairment or psychiatric disorder frequently constitute the initial clinical symptoms of patients with CSF1R-microglial encephalopathy. Motor dysfunction may frequently develop with the progression of the disease, clinically manifesting as bradykinesia and postural instability due to parkinsonism (Konno et al., 2017; Ikeuchi et al., 2018; Zhuang et al., 2020), spastic gait because of pyramidal injury (Konno et al., 2017), or a combination of both (Kraya et al., 2019). The proband in the first family developed mixed gait disturbance due to pyramidal and extrapyramidal damage, while his sister only exhibited bradykinesia, suggesting variability in the presentations of motor dysfunction in CSF1R-microglial encephalopathy. These cognitive, psychiatric, and motor features are characteristic of encephalopathic changes. However, evaluation of the accuracy of the established diagnostic criteria using an independent multicenter cohort in 2021 showed that the ALSP criteria possessed overall limited sensitivity with modest specificity (Ayrignac et al., 2022). Patients suspected of having CSF1R-microglial encephalopathy should undergo comprehensive brain MRI and genetic testing using panel or exome sequence analyses. However, the application of *CSF1R* gene testing may be restricted in clinical practice due to its cost and availability. Moreover, some individuals may carry *CSF1R* mutations and remain asymptomatic, possibly because they may not have reached the age of onset or due to incomplete penetrance. In the first family, patient Ⅲ-11 with a *CSF1R* mutation was older than the proband but remained asymptomatic at the age of 45 years. Moreover, almost no specific white matter change was observed on structural brain MRI. In the second family, patient Ⅱ-4, the proband’s mother, did not complain of any significant clinical symptoms. These findings were similar to that of previous studies. Karle et al. reported that the 69-year-old father of their index patient carried a *CSF1R* mutation but was clinically unaffected, with few non-specific white matter lesions on brain MRI, presumably due to the existing classical vascular risk factors (Karle et al., 2013). Another study demonstrated that the proband’s mother retained functional independence at the age of 79 years, despite having a psychiatric illness for more than 20 years after the onset of psychiatric symptoms (Rademakers et al., 2011). The above-mentioned findings suggest incomplete penetrance or marked intrafamilial variability in CSF1R-microglial encephalopathy. We speculated that the identified variant might be subject to genetic modifiers that prevent its manifestation. The possibility that these unaffected individuals may develop symptoms at an older age cannot be disregarded, considering that the age of onset has been noted even at 78 years (Chitu et al., 2021). It is noteworthy that although only a few non-specific white matter lesions were observed on structural brain MRI in patient III-11, pCASL demonstrated a marked decrease in the CBF in the bilateral periventricular areas, corona radiata, and corpus callosum that was not captured by the structural brain MRI. It has been recently reported that CBF values measured using pCASL are more sensitive than those obtained with conventional structural MRI to evaluate the blood flow and neural activity in numerous neurological conditions, including AD (Leeuwis et al., 2017), stroke (Bhattacharjee et al., 2021), and brain tumor (Cohen et al., 2020). However, pCASL has seldom been used for imaging leukoencephalopathies and even less so for CSF1R-microglial encephalopathy. We speculated that the incomplete penetrance, manifesting as slight or even the absence of white matter lesions, may be attributed to the low sensitivity of conventional structural brain MRI. Therefore, our study recommends that a pCASL pattern-based approach should be adopted since it enables early detection of areas with decreased perfusion.

In conclusion, we developed the concept “CSF1R-microglial encephalopathy” that encompasses the genetic, pathological, and clinical characteristics and their respective relationships to describe the various phenotypic spectrum of a unified disease due to *CSF1R* mutations better. We identified two novel splicing mutations (c.2319+1C>A) in intron 20 and (c.1858+5G>A) in intron 13 of *CSF1R* as the cause of CSF1R-microglial encephalopathy, and firstly highlighted the potential of the pCASL technique to enhance the clinical diagnostic accuracy of CSF1R-microglial encephalopathy, further broadening the genetic and radiological spectrum of this condition. Neurologists should consider CSF1R-microglial encephalopathy when a patient exhibits early-onset rapid progressive dementia with symmetrical changes and persistent diffusion restriction in the deep white matter because the genetic spectrum of mutations is yet to be fully elucidated. Future functional research is needed to elucidate the pathogenicity of these two novel intronic mutations of *CSF1R*. Prospective studies should be developed to identify the sensitivity and specificity of pCASL technique to diagnose the CSF1R-microglial encephalopathy.

## Data Availability

The datasets presented in this study can be found in online repositories. The names of the repository/repositories and accession number(s) can be found below: https://www.ncbi.nlm.nih.gov/, PRJNA780236.
